# Annotations

**Published:** 1905-03-11

**Authors:** 


### Annotations.

### The Clergy and Medical Fees.

An animated controversy is going on in the *Church Times* on the subject of the clergy and medical fees. A "Suffolk Priest" set the ball rolling by complaining that through long years of a struggle to bring up a large family on slender means, both as curate and rector, no medical man had ever offered to let him off any fee, though his needy circumstances are well known. He added that an Irish clergyman had told him that in the Emerald Isle "medical men never thought of taking fees from the clergy." A medical man now asks the Suffolk priest why he should be expected to work for nothing; and another, who says that the question is worth ventilating, states that during the nearly 20 years he has been in practice he has always attended his own parish priest, his curates, and their families gratuitously, but that the struggle of the country doctor to make two ends meet is often as severe as that of the country parson. One clergyman, narrating his own experience, testifies to the extreme generosity of medical men, and affirms that again and again both the highest specialists and the humblest general practitioners have absolutely refused his proffered cheque; while a second, entering "an emphatic protest against a Suffolk priest's slur on medical men," also attests their liberality, but asks on what ground the clergy claim to call for the professional skill of a medical man, which he has cultivated at large original outlay and carries on at great present expense, without rendering an equivalent. We dissent from the view of this last correspondent that it is a slur on any professional man to accuse him of taking the fees which he has earned. If he remits them, it is an act of grace. We believe that these acts of grace are more frequently performed by medical men than by any other class. But people have no more right to expect a doctor to attend them gratuitously than a baker to send them bread without payment; and some of the clergy are better able to pay fees than some medical men are to go without them. If a parson obtains relief or exemption it should not be because of his calling, but because he is poor; while to rail against a man belonging to another profession because he accepts what is due to him is not only unreasonable, but is in contravention of the golden rule which the clergy are specially supposed to inculcate.

### Anti-Malarial Measures in Africa.

The Liverpool School of Tropical Medicine has just put forth a Memoir (Number XIV.) on the results which have followed from the teaching of their expeditions respectively at Bathurst, Conakry, and Freetown, all in North-West Africa, where Conakry is in French territory and the other towns dealt with are in English. Conakry was established by the French distinctly as a commercial rival to Freetown, and is not only built upon an island which secures to it many sanitary advantages, but is also planned and constructed entirely on modern lines, so as to be free from many of the difficulties, in the way of defective and ruinous buildings, by which the work of the authorities is impeded in other localities upon the coast. Notwithstanding these difficulties, the greater care which has been taken in the English settlements with regard to the destruction of anopheles larvæ has been fruitful of good results; insomuch that, while malarious and blackwater fevers have been frequent at Conakry, they have been almost entirely absent from Bathurst, and comparatively rare even at Freetown, where the improvement in the health of the European population is so marked that the local medical officer hesitates to ascribe the whole of it to the reforms, and speaks doubtfully of the possible effect, in the same direction, of a large number of new arrivals. But there seems to be no immediate prospect of materially diminishing the disease among the natives, so that the blood of their children is likely to remain for some time a source of infection to such specimens of anopheles as are permitted to come to maturity; and on this ground, if on no other, the destruction of the insects will not be likely for the present to render it safe to sleep without the protection of a carefully arranged net.

### Teachers and Examiners.

Professor Arthur Crossley, of the Pharmaceutical Society, in a review of the regulations governing the training of pharmacists in Germany, emphasises a comparison which, in relation to thoroughness and efficiency, is by no means to the advantage of Great Britain. In dealing with the subject of the examinations he points out that in Germany these are conducted by the teachers and he is enthusiastic in favour of this method. He considers it ridiculous to suppose that an examiner, having no previous acquaintance with a student, can, in the course of a comparatively brief interview, do justice to attainments which have been acquired by work extending over several years. And against the suggestion that the plan he favours must mean that the candidate is familiar in advance with what he is likely to be asked, he sensibly remarks that even on a single subject no student can be expected to have a really exhaustive knowledge. Again, it has to be remembered that the German professor largely depends on the fees paid by students on entering his laboratory or class-room, and the number of these is necessarily in direct proportion to his reputation as an efficient teacher. Hence, even for selfish reasons, he cannot afford to send into the world men bearing his hall-mark who have failed properly to use their opportunities for acquiring knowledge. Granted a sufficiently long and properly directed curriculum, there is a growing tendency in favour of the teacher taking a leading part in the conduct of the qualifying examination. It means in practice that the examination is ruled by the teaching and not the teaching by the examination. Hence the teacher is free to conduct his students along lines which train the mind and develop natural capacity instead of having constantly to consider the wants of a more or less arbitrarily constituted board of examiners. In the Scottish Universities there is associated with the teacher in the conduct of the examinations an independent expert, and this plan is also being adopted by the newer universities in England. Possibly in some remote future even the London medical corporations may recognise that such a plan favours true education.

